# Transumbilical single-incision laparoscopic-assisted removal of gastric trichobezoars in children: a technical refinement and feasibility study

**DOI:** 10.1186/s12893-026-03628-2

**Published:** 2026-05-25

**Authors:** Ahmed Maher, Almoutaz A Eltayeb, Hussein Ibrahim

**Affiliations:** Department of Pediatric Surgery, Faculty of Medicine, Assiut University, Assiut, Egypt

**Keywords:** Gastric trichobezoar, Laparoscopic-assisted surgery, Trichotillomania, Cosmetic outcomes, Low-resource settings

## Abstract

**Background:**

Gastric trichobezoars are rare in children and adolescents and are often associated with psychiatric disorders such as trichotillomania and trichophagia. While surgical removal remains the definitive treatment for large bezoars, optimal management must consider not only technical success but also cosmetic outcomes, psychosocial impact, and feasibility in low-resource settings. The aim of the study was to evaluate the safety, feasiblity, and cosmetic of a transumbilical single-incision laparoscopic-assisted technique for gastric trichobezoar removal in children.

**Methods:**

We conducted a retrospective review of pediatric patients who underwent transumbilical single-incision laparoscopic-assisted removal of gastric trichobezoars. Demographic, characteristics, psychiatric comorbidity, clinical presentation, imaging findings, operative details, and postoperative outcomes were analyzed.

**Results:**

Five pediatric patients (median age 12 years; range, 9–14 years) were included. Psychiatric comorbidity was present in four of five patients (80%). Complete bezoar removal was achieved in all cases without conversion to open surgery. The median operative time was 120 min (range, 90–150 min). One patient developed a superficial wound infection that resolved with conservative management. The median hospital stay was 3 days (range, 2–4 days). Umbilical scars were well concealed in all patients, with satisfactory early cosmetic outcomes.

**Conclusion:**

The transumbilical laparoscopic-assisted approach was safe and effective, with concealed scarring and demonstrated feasibility using standard surgical instruments.

## Background

Gastric trichobezoars are uncommon condition in pediatric patients and are frequently associated with trichotillomania and trichophagia [1]. The clinical presentation is often nonspecific and may include abdominal pain, vomiting, early satiety, anemia, and weight loss. Extension of the bezoar into the small bowel can result in Rapunzel syndrome, which may lead to intestinal obstruction or perforation [2, 3].

Endoscopic management has been proposed as a minimally invasive option; however, its effectiveness in pediatric patients with large trichobezoars is limited by the dense, matted structure of hair masses. Reported success rates remain below 5%, and endoscopic extraction carries an increased risk of mucosal injury and gastric perforation [4, 5].

Open laparotomy remains a reliable surgical approach because it permits complete bezoar extraction and comprehensive gastrointestinal inspection. Nevertheless, it is associated with increased postoperative pain, prolonged hospitalization, wound-related morbidity, and visible abdominal scarring [6]. These disadvantages are particularly relevant in psychologically vulnerable pediatric patients, in whom cosmetic outcomes may significantly influence body image and postoperative recovery [7–9].

Minimally invasive alternatives have therefore gained increasing interest. Pure laparoscopic techniques offer improved cosmetic outcomes but are technically demanding, time-consuming, and associated with a risk of peritoneal contamination during intracorporeal fragmentation [5, 10]. Laparoscopic-assisted approaches aim to balance safety, efficiency, cosmetic benefit, and feasibility, particularly in low-resource settings where access to advanced laparoscopic technology may be limited.

In this study, we present five pediatric patients who underwent transumbilical single-incision laparoscopic-assisted removal of gastric trichobezoars.

## Methods

### Study design and patients

This retrospective study included pediatric patients diagnosed with gastric trichobezoars who underwent transumbilical single-incision laparoscopic-assisted removal between 2017 and 2024.

Diagnosis was established based on clinical presentation, imaging findings, and upper gastrointestinal endoscopic evaluation when available. At our institution, the transumbilical single-incision laparoscopic-assisted approach has become the preferred technique for the management of large gastric trichobezoars in clinically stable pediatric patients. Open surgery is reserved for patients presenting with hemodynamic instability, gastric perforation, generalized peritonitis, or extensive distal small-bowel involvement.

Inclusion criteria comprised pediatric patients with gastric trichobezoars requiring operative removal who were treated using the described transumbilical laparoscopic-assisted approach during the study period. Exclusion criteria included patients presenting with generalized peritonitis or gastric perforation requiring laparotomy, as well as cases with extensive distal small-bowel bezoars in whom primary open surgery was considered more appropriate.

Clinical presentation at admission included abdominal pain, vomiting, early satiety, abdominal distension, and/or a palpable epigastric mass. All patients underwent preoperative abdominal ultrasonography and computed tomography to confirm the diagnosis, assess bezoar size, and evaluate possible extension beyond the stomach.

Radiological diagnosis of Rapunzel syndrome was established in two patients based on computed tomography findings demonstrating extension of the gastric trichobezoar beyond the pylorus into the duodenum, with associated gastric and proximal duodenal distension. No distal small-bowel obstruction was identified on preoperative imaging in these cases.

### Data collection

Collected variables included age, sex, psychiatric history, abdominal examination findings, presence of Rapunzel syndrome, imaging characteristics, operative time, postoperative complications, and length of hospital stay.

### Surgical technique

Under general anesthesia, the patient was placed in the supine position. A vertical transumbilical skin incision measuring approximately 2–3 cm was made, with careful preservation of the umbilical contour.

**Fig. 1:**
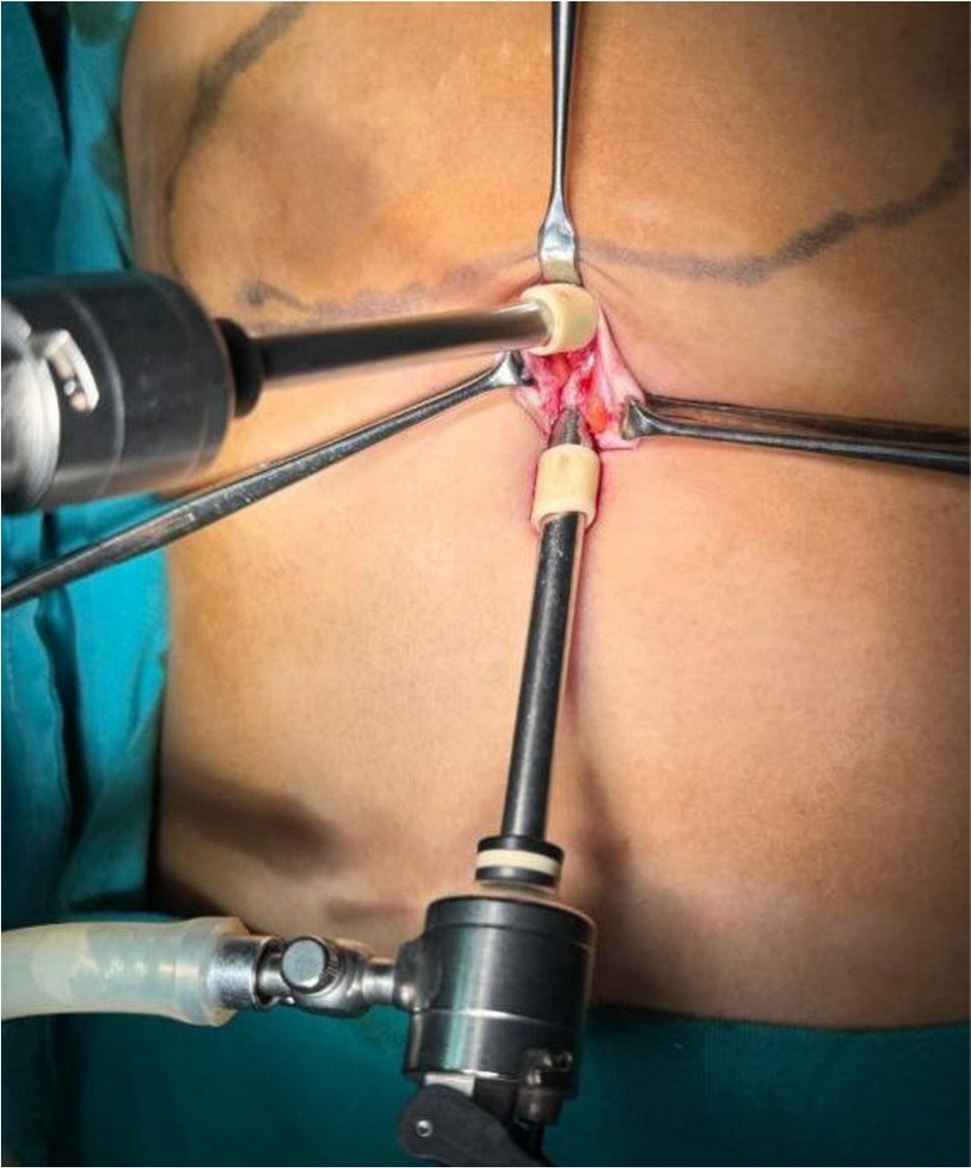
Transumbilical access and port placement.A single vertical transumbilical incision is used to introduce two 5-mm trocars through separate fascial punctures within the same incision. The inferior port accommodates the laparoscopic camera, while the superior port is used for the working instrument

A 5-mm trocar for the laparoscope was introduced through the natural umbilical fascial defect using an open cut-down technique. When necessary, the fascial opening was slightly enlarged to accommodate the trocar. Pneumoperitoneum was established, and a 5-mm laparoscope was inserted. Under direct vision, a second 5-mm trocar for an atraumatic grasper or working instrument was placed through the linea alba, superior to the first port, via a separate fascial puncture within the same umbilical incision (Fig. 1).

The stomach was identified and gently grasped at the antrum, then drawn toward the umbilical port. The fascial bridge between the two trocars was divided longitudinally, allowing the stomach wall to be exteriorized through the umbilical incision. Both trocars and the laparoscope were temporarily removed.

**Fig. 2:**
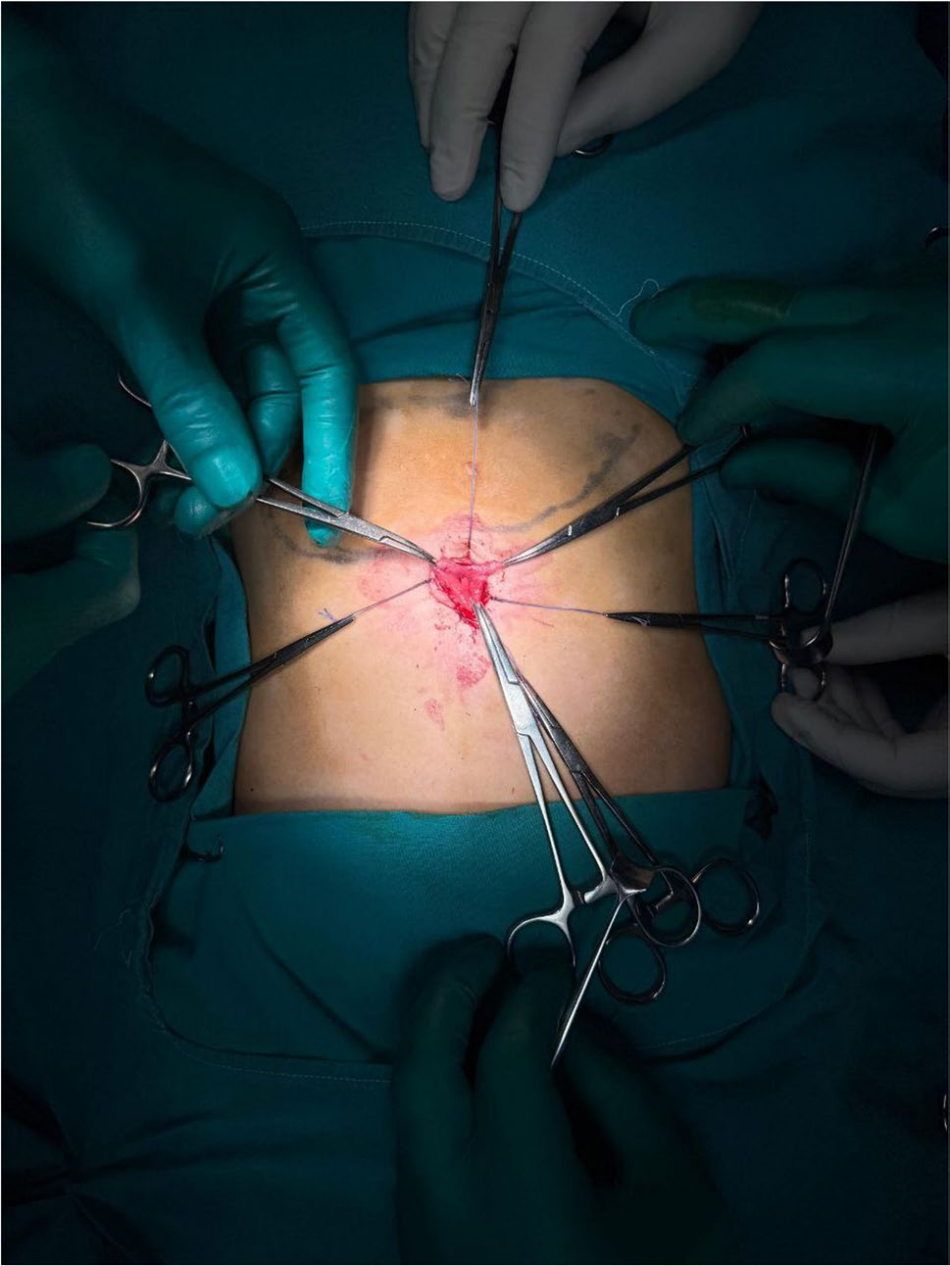
Exteriorization and fixation of the stomach wall. Following laparoscopic mobilization, the stomach is exteriorized through the umbilical incision and secured to the abdominal wall using stay sutures. An anterior gastrostomy is created with circumferential fixation to the skin to isolate the operative field and prevent peritoneal contamination

The anterior gastric wall was secured to the abdominal wall using four stay sutures to ensure stability and complete isolation of the operative field. A controlled 2–2.5 cm gastrostomy was created on the anterior gastric wall using diathermy. The edges of the gastrostomy were sutured circumferentially to the skin, effectively sealing the stomach to the abdominal wall and preventing peritoneal contamination (Fig. 2).

**Fig. 3:**
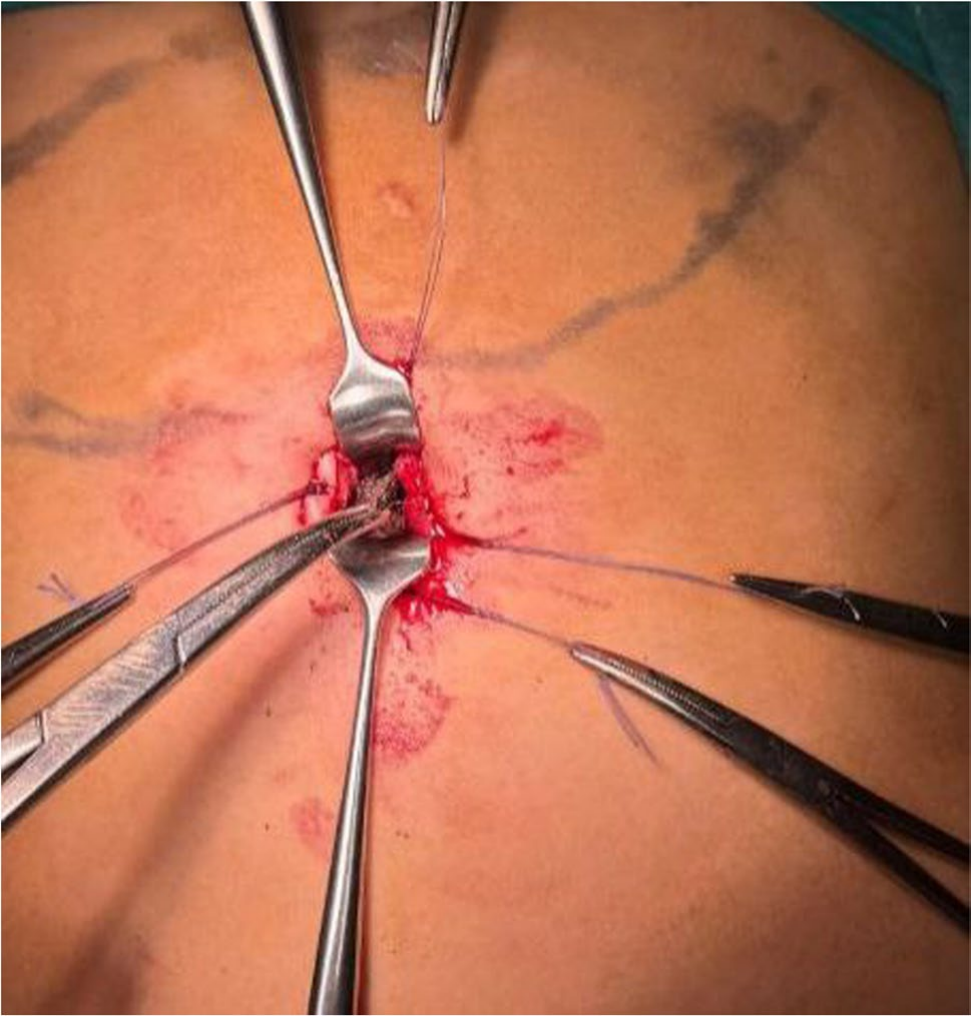
Piecemeal extraction of the gastric trichobezoar. The trichobezoar is removed extracorporeally through the gastrostomy using standard instruments, with care taken to avoid spillage

The trichobezoar was removed piecemeal using artery forceps or a Rampley sponge holder, with meticulous care taken to avoid spillage (Fig. 3). After extraction, the laparoscope was introduced through the gastrostomy to inspect the gastric cavity for residual fragments. Pneumoperitoneum was then re-established, and the entire small intestine was examined laparoscopically to exclude distal bezoars.

Systematic inspection of the small intestine was performed by sequential “running” of the bowel from the ligament of Treitz to the ileocecal junction using an atraumatic grasper, with the bowel gently manipulated and aligned against the abdominal wall under direct laparoscopic visualization.

Two patients were diagnosed preoperatively with Rapunzel syndrome. In both cases, intraoperative findings confirmed extension of the trichobezoar beyond the pylorus into the duodenum, without distal small-bowel extension. Following exteriorization of the stomach through the umbilical incision, complete bezoar removal was achieved solely via gastrotomy, and no additional enterotomy was required.

Following thorough irrigation, the gastrostomy was closed in two layers using absorbable polydioxanone sutures. The fascia and skin were closed anatomically, restoring the umbilical contour.

## Results

Five pediatric patients (four females and one male) with a median age of 12 years (range, 9–14 years) were included in the study. Psychiatric comorbidity, predominantly trichotillomania, was identified in four patients; all were referred for psychiatric evaluation and counseling as part of routine clinical care. Two patients demonstrated radiological evidence of Rapunzel syndrome. Characteristics of patients are shown in Table 1.

**Table 1:** Demographic, clinical, imaging, and perioperative characteristics of the study cohort

Patients	Age (years)	Sex	Psychiatric morbidity	Abdominal examination	Imaging findings	Rapunzel syndrome	Upper GI endoscopy	Operative time(min.)	Hospital stay (days)
1	12	Female	Trichotillomania; psychosocial stressors	Distended abdomen with firm epigastric mass	Gastric trichobezoar ~ 15 × 10 cm	No	Not amenable	120	2
2	14	Female	Trichotillomania with alopecia	Large smooth tender epigastric mass	Distended stomach and duodenum ~ 12 × 15 cm	Yes	Not available	150	4
3	10	Female	Trichotillomania	Large mass occupying upper abdomen	Large heterogeneous mass extending to duodenum	Yes	Not amenable	130	3
4	13	Female	Trichotillomania with alopecia	Firm epigastric mass	Gastric trichobezoar ~ 10 × 12 cm	No	Not available	110	4
5	9	Male	Psychosocial stressors	Unremarkable	Gastric trichobezoar ~ 10 × 10 cm	No	Not amenable	90	3

Preoperative ultrasonography and computed tomography revealed large intragastric trichobezoars ranging from 10 to 15 cm in maximal diameter. Upper gastrointestinal endoscopy, when performed, confirmed that the bezoars were not amenable to endoscopic extraction.

### Patient characteristics

### Operative and postoperative outcomes

The median operative time was 120 min (range, 90–150 min). Complete bezoar removal was achieved in all cases without conversion to open surgery or intra-abdominal contamination. One patient developed a superficial wound infection, which resolved with conservative management. Oral feeding was initiated on the first postoperative day.

The median length of hospital stay was 3 days (range, 2–4 days). No reoperations or readmissions occurred. Umbilical scars were effectively concealed in all patients, and no early cosmetic or body-image concerns were reported during follow-up.

Patients were followed for a median duration of 4 years (range, 2–8 years). During follow-up, all patients were regularly reviewed in the psychiatric clinic as part of multidisciplinary care. No late surgical complications or trichobezoar recurrence were documented during the available follow-up period.

## Discussion

The predominance of psychiatric comorbidity among our patients reflects the well-recognized association between gastric trichobezoars and trichotillomania or trichophagia, underscoring the need for surgical strategies that also consider psychosocial impact [1]. In this particularly vulnerable population, the objectives of surgical management extend beyond successful bezoar extraction to include psychosocial well-being, preservation of body image, and facilitation of psychological recovery.

Endoscopic management has been proposed as a minimally invasive alternative; however, its role in pediatric gastric trichobezoars remains limited. Large trichobezoars are resistant to endoscopic extraction because of their dense, matted composition, and fewer than 5% can be successfully removed endoscopically [4, 5]. Consequently, endoscopy alone is generally inadequate as definitive therapy in pediatric patients with large gastric trichobezoars.

Open laparotomy has traditionally been considered the standard surgical approach, owing to its reliable extraction and comprehensive gastrointestinal inspection, particularly in cases of Rapunzel syndrome [6]. Despite its effectiveness, open surgery is associated with increased postoperative pain, prolonged hospitalization, higher wound-related morbidity, and significant abdominal scarring. These disadvantages are especially relevant in children and adolescents with psychiatric disorders, in whom visible scars may exacerbate psychosocial distress [7–9].

Pure laparoscopic techniques have been reported with the aim of reducing surgical trauma and improving cosmetic outcomes. However, complete laparoscopic removal of large gastric trichobezoars is technically demanding and frequently associated with prolonged operative times due to intracorporeal fragmentation. Moreover, manipulation of hair masses within the peritoneal cavity carries a risk of contamination and postoperative infectious complications [5, 10]. These limitations have hindered the widespread adoption of fully laparoscopic approaches, particularly in centers with limited advanced laparoscopic expertise or resources.

Laparoscopic-assisted techniques were developed to overcome these challenges by combining laparoscopic mobilization with extracorporeal extraction. In 1998, the first laparoscopic-assisted removal of a gastric bezoar was reported by Nirasawa [11], followed by several successful reports using similar approaches [10, 12]. However, many previously described techniques rely on additional incisions or require specialized wound protection devices, which may compromise cosmetic outcomes and limit applicability in resource-constrained environments [13–17].

The technique presented in the present study represents a technical refinement that simultaneously addresses cosmetic, safety, and resource-related considerations. The use of a single concealed transumbilical incision results in minimal visible scarring and preservation of the normal umbilical contour. This cosmetic advantage is not merely aesthetic; rather, it carries substantial psychosocial importance for pediatric patients with underlying psychiatric disorders, in whom avoidance of stigmatizing scars may positively influence body image, self-perception, and postoperative psychological outcomes.

Equally important, this technique demonstrates high feasibility and reproducibility in low-resource settings. The procedure relies exclusively on standard laparoscopic instruments and basic suturing techniques, without the need for advanced energy devices, endoscopic staplers, or commercially available wound protectors. Secure fixation of the stomach to the abdominal wall before gastrostomy creation provides effective isolation of the operative field and minimizes peritoneal contamination. This contamination-control strategy is simple, cost-effective, and easily reproducible, making it particularly suitable for institutions with limited financial and technological resources.

Routine intragastric visualization combined with laparoscopic inspection of the entire small bowel further strengthens the technique by ensuring complete bezoar clearance and reducing the risk of missed distal fragments, a complication reported in previous series [18]. Importantly, these steps can be performed using basic laparoscopic equipment, reinforcing the adaptability of the approach across low-resource surgical environments.

The median operative time of 120 min observed in the present series lies within the lower range of operative durations reported for minimally invasive management of gastric trichobezoars. A review of published laparoscopic and laparoscopic-assisted series reporting operative duration demonstrates a median operative time of approximately 3 h, reflecting the technical complexity of the procedure and the frequent need for piecemeal extraction [10, 11, 19–21]. Conventional open gastrotomy via laparotomy has historically been the standard approach for large or complicated trichobezoars and allows direct access to the stomach with efficient bezoar extraction and bowel inspection; operative times reported for open surgery are generally shorter in some series, although detailed and consistent reporting is limited. Despite the potential for longer operative duration, minimally invasive and laparoscopic-assisted approaches offer important advantages in terms of reduced wound morbidity and improved cosmetic outcomes, which are particularly relevant in pediatric patients, including those with underlying psychiatric vulnerability. Direct comparison, however, is limited by differences in patient selection and study design.

This study has several limitations. Its retrospective design and small sample size limit generalizability. Cosmetic outcomes were assessed subjectively based on clinical inspection during follow-up and patient or guardian satisfaction, without the use of validated scar assessment scales. Although long-term follow-up was conducted in the psychiatric clinic, psychological outcomes and relapse were not evaluated using standardized or validated instruments, limiting objective assessment of psychosocial impact. In addition, the absence of a comparative cohort restricts direct comparison with alternative surgical approaches.

Accordingly, the present work was not designed as a hypothesis-testing or outcome-comparison study, but rather as a feasibility and technical refinement report. The primary aim was to demonstrate the safety, reproducibility, contamination control, and cosmetic advantages of the described technique using standard laparoscopic instruments.

## Conclusions

Transumbilical single-incision laparoscopic-assisted removal of gastric trichobezoars is a safe and effective technique that provides favorable cosmetic outcomes using standard surgical instruments. It represents a practical option for pediatric patients, particularly in resource-limited settings.

## Data Availability

The datasets used and/or analyzed during the current study are available from the corresponding author upon reasonable request.
